# Shaped by Their Environment: Variation in Blue Whale Morphology across Three Productive Coastal Ecosystems

**DOI:** 10.1093/iob/obad039

**Published:** 2023-11-20

**Authors:** D R Barlow, K C Bierlich, W K Oestreich, G Chiang, J W Durban, J A Goldbogen, D W Johnston, M S Leslie, M J Moore, J P Ryan, L G Torres

**Affiliations:** Geospatial Ecology of Marine Megafauna Lab, Marine Mammal Institute, Department of Fisheries, Wildlife, and Conservation Sciences, Oregon State University, Newport, OR 97365, USA; Geospatial Ecology of Marine Megafauna Lab, Marine Mammal Institute, Department of Fisheries, Wildlife, and Conservation Sciences, Oregon State University, Newport, OR 97365, USA; Monterey Bay Aquarium Research Institute, Moss Landing, CA 95039, USA; Centro de Investigación para la Sustentabilidad (CIS) & Departamento de Ecología y Biodiversidad, Universidad Andrés Bello, 8370251, Santiago, Chile; Marine Mammal Institute, Oregon State University, Newport, OR 97365, USA; Hopkins Marine Station, Department of Biology, Stanford University, Pacific Grove, CA 93950, USA; Division of Marine Science and Conservation, Nicholas School of the Environment, Duke University Marine Laboratory, Beaufort, NC 28516, USA; National Climate Adaptation Science Center, United States Geological Survey, Reston, VA 20192, USA; Biology Department, Woods Hole Oceanographic Institution, Woods Hole, MA 02543, USA; Monterey Bay Aquarium Research Institute, Moss Landing, CA 95039, USA; Geospatial Ecology of Marine Megafauna Lab, Marine Mammal Institute, Department of Fisheries, Wildlife, and Conservation Sciences, Oregon State University, Newport, OR 97365, USA

## Abstract

Species ecology and life history patterns are often reflected in animal morphology. Blue whales are globally distributed, with distinct populations that feed in different productive coastal regions worldwide. Thus, they provide an opportunity to investigate how regional ecosystem characteristics may drive morphological differences within a species. Here, we compare physical and biological oceanography of three different blue whale foraging grounds: (1) Monterey Bay, California, USA; (2) the South Taranaki Bight (STB), Aotearoa New Zealand; and (3) the Corcovado Gulf, Chile. Additionally, we compare the morphology of blue whales from these regions using unoccupied aircraft imagery. Monterey Bay and the Corcovado Gulf are seasonally productive and support the migratory life history strategy of the Eastern North Pacific (ENP) and Chilean blue whale populations, respectively. In contrast, the New Zealand blue whale population remains in the less productive STB year-round. All three populations were indistinguishable in total body length. However, New Zealand blue whales were in significantly higher body condition despite lower regional productivity, potentially attributable to their non-migratory strategy that facilitates lower risk of spatiotemporal misalignment with more consistently available foraging opportunities. Alternatively, the migratory strategy of the ENP and Chilean populations may be successful when their presence on the foraging grounds temporally aligns with abundant prey availability. We document differences in skull and fluke morphology between populations, which may relate to different feeding behaviors adapted to region-specific prey and habitat characteristics. These morphological features may represent a trade-off between maneuverability for prey capture and efficient long-distance migration. As oceanographic patterns shift relative to long-term means under climate change, these blue whale populations may show different vulnerabilities due to differences in migratory phenology and feeding behavior between regions.

**Spanish abstract** La ecología y patrones de historia de vida de las especies a menudo se reflejan en la morfología animal. Las ballenas azules están distribuidas globalmente, con poblaciones separadas que se alimentan en diferentes regiones costeras productivas de todo el mundo. Por lo tanto, brindan la oportunidad de investigar cómo las características regionales de los ecosistemas pueden impulsar diferencias morfológicas dentro de una especie. Aquí, comparamos la oceanografía física y biológica de tres zonas de alimentación diferentes de la ballena azul: (1) Bahía de Monterey, California, EE. UU., (2) Bahía del sur de Taranaki (BST), Nueva Zelanda, y (3) Golfo de Corcovado, Chile. Adicionalmente, comparamos la morfología de las ballenas azules de estas regiones utilizando imágenes de aeronaves no tripuladas. La Bahía de Monterey y el Golfo de Corcovado son estacionalmente productivos y apoyan la estrategia migratoria de la historia de vida de las poblaciones de ballena azul chilena y del Pacífico Norte Oriental (PNO), respectivamente. Por el contrario, la población de ballena azul de Nueva Zelanda permanece en la menos productiva BST durante todo el año. Las tres poblaciones eran indistinguibles en cuanto a la longitud corporal total. Sin embargo, las ballenas azules de Nueva Zelanda tenían una condición corporal significativamente mayor a pesar de una menor productividad regional, potencialmente atribuible a su estrategia no migratoria que facilita un menor riesgo de desalineación espaciotemporal con oportunidades de alimentación disponibles de manera más consistente. Alternativamente, la estrategia migratoria de las poblaciones de ballenas PNO y chilena puede tener éxito cuando su presencia en las zonas de alimentación se alinea temporalmente con la abundante disponibilidad de presas. Documentamos diferencias en la morfología del cráneo y la aleta caudal entre poblaciones, que pueden estar relacionadas con diferentes comportamientos de alimentación adaptados a las características de hábitat y presas específicas para cada región. Estas características morfológicas pueden representar una compensación entre la maniobrabilidad para la captura de presas y una migración eficiente a larga distancia. A medida que los patrones oceanográficos cambian en términos de mediano a largo plazo debido al cambio climático, estas poblaciones de ballenas azules pueden mostrar diferentes vulnerabilidades debido a diferencias en la fenología migratoria y el comportamiento de alimentación entre regiones.

## Introduction

The shape and size of organisms often reflects their ecological role (Banavar et al. 2014). For example, animal morphology may be influenced by behavior, habitat, diet, or trophic level. This concept that form follows function has been illustrated across a wide range of vertebrate taxa, whereby adaptations of an animal to their environment are reflected in their morphology (Webb 1984; Aerts et al. 2000; Woodward et al. 2006; Pigot et al. 2020). Even within vertebrate species, morphology can differ between geographic regions and across environmental gradients (Chapman et al. 2015; Grilli et al. 2017; Shuai et al. 2018). Therefore, understanding evolutionary and ecological differences among subspecies and populations could be enhanced by investigating the interplay between ecosystem characteristics and animal morphology.

Blue whales (*Balaenoptera musculus*) are a globally distributed, mobile marine vertebrate with extreme energetic demands due to their massive size (Williams et al. 2001). Their lifestyle necessitates efficient exploitation of densely aggregated but ephemeral prey patches. They employ a foraging behavior known as lunge feeding, consisting of intermittent, high-speed engulfment events enabled by fast acceleration, an extensible buccal cavity capable of holding large volumes of prey-laden water, and filtration through baleen plates (Goldbogen et al. 2017). This specialization has facilitated an evolutionary pathway to extreme body sizes, whereby size is essentially constrained by prey availability (Goldbogen et al. 2012; Goldbogen et al. 2019). Furthermore, blue whales have a streamlined morphology, built for both efficient long-distance movements and maneuverability for prey capture (Woodward et al. 2006; Gough et al. 2019; Segre et al. 2022).

**Fig. 1:**
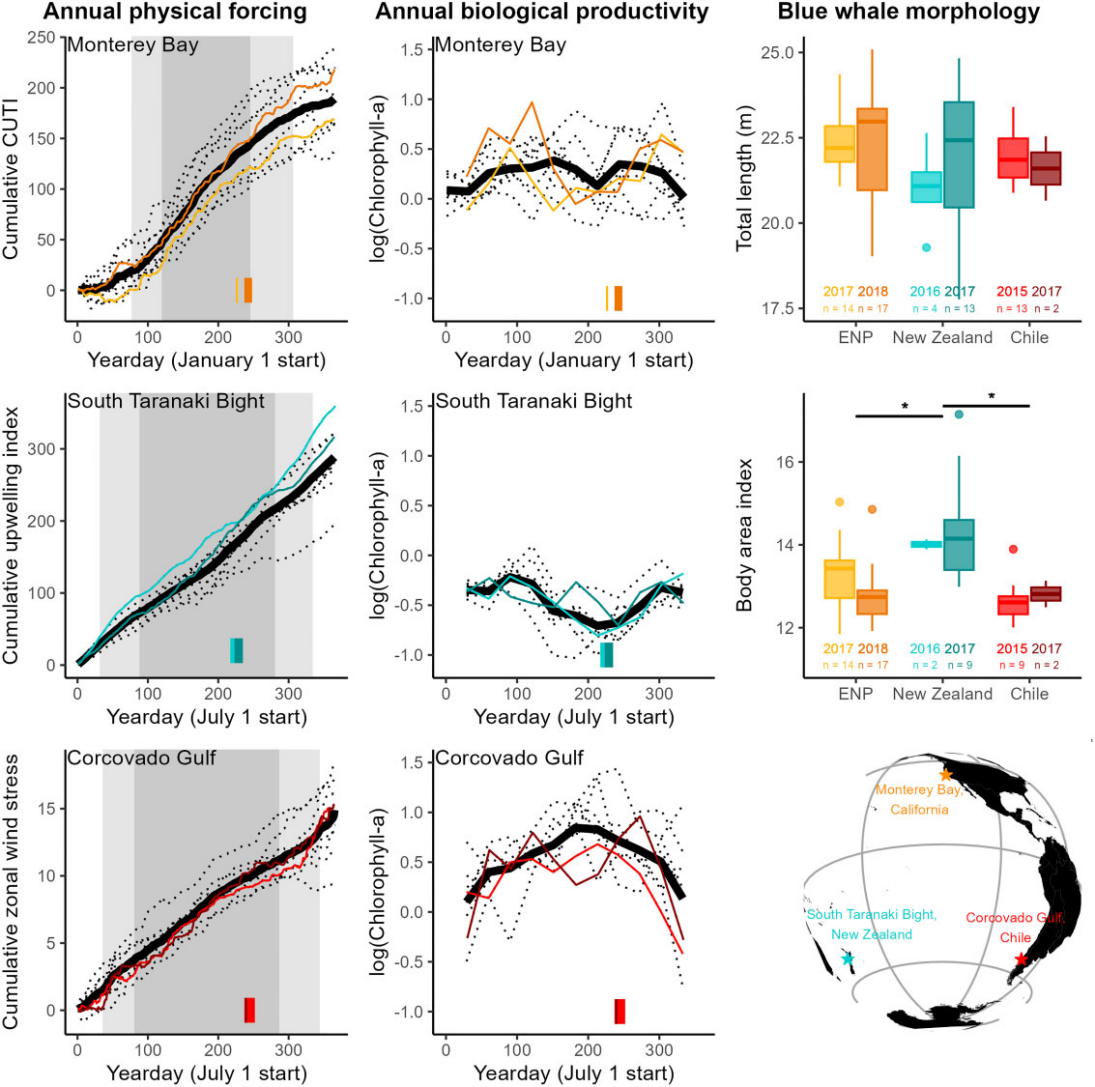
Left column: cumulative physical forcing metric for each region (coastal upwelling transport index, upwelling index, and zonal wind stress, respectively), with shading denoting the 50% (dark gray) and 90% (light gray) annual accumulation windows. Center column: biological productivity in each system. Each dotted line represents a different year. The dark black line represents the mean. Colored lines correspond to years morphological data were collected; corresponding sampling periods are denoted by colored ribbons along the *x*-axis. Right column: box plots show group median, interquartile range (IQR, box), maximum and minimum 1.5*IQR (vertical lines). Black bars above box plots with asterisks indicate statistically significant differences based on Monte Carlo ANOVA results.

Most baleen whales are capital breeders that make long-distance migrations between foraging grounds and wintering areas, depending on seasonally available prey to increase fat reserves that support the cost of reproduction during a defined breeding season (Lockyer 1981). However, not all baleen whales fit this paradigm (Geijer et al. 2016). Even within our focal species, different blue whale populations fall on a spectrum of life history strategies ranging from stereotyped migration (Double et al. 2014; Abrahms et al. 2019) to year-round residency (Ilangakoon and Sathasivam 2012; Barlow et al. 2018). In this study, we focus on three blue whale populations, representing three subspecies, that rely on different foraging grounds (Fig. 1) and exhibit different migration patterns, target prey species, and behaviors. Whether these populations show distinct morphological adaptations to regional physical oceanography, primary productivity, and ecology of prey remains unknown. We postulate that distinct ecosystem dynamics shape the morphology of blue whale populations to be optimally suited for the environments they inhabit.

The Eastern North Pacific (ENP) blue whale population (*B. m. musculus*) feeds seasonally off the west coast of North America, including the productive waters of Monterey Bay, California, USA, and their population size is estimated to be stable at approximately 2000 individuals (Calambokidis and Barlow 2020). ENP blue whales migrate between these foraging grounds in summer and fall and lower-latitude wintering areas near Baja California and the Costa Rica Dome in winter and spring (Bailey et al. 2009). On the foraging grounds, upwelling-favorable winds drive primary productivity and lead to seasonally abundant prey, *Thysanoessa spinifera* and *Euphausia pacifica* krill (Croll et al. 2005).

The New Zealand blue whale population that feeds in the South Taranaki Bight region (STB) of Aotearoa New Zealand, consists of approximately 700 individuals, and the population trend is unknown (Barlow et al. 2018). This population is within the pygmy blue whale subspecies (*B. m. brevicauda*), named for their shorter length than Antarctic blue whales (Ichihara 1966). In contrast to the ENP population, New Zealand blue whales are present in the STB year-round, and likely rely on the area for both feeding and breeding (Barlow et al. 2023). The STB is home to a coastal upwelling system, where pulsed wind events drive an upwelling plume (Barlow et al. 2021) that is present during all times of year (Chiswell et al. 2017), supporting aggregations of *Nyctiphanes australis* krill that are target prey for blue whales (Barlow et al. 2020).

The Chilean blue whale population that feeds in the waters off Northern Chilean Patagonia belongs to a third, yet-unnamed subspecies (Committee on Taxonomy 2021). The population size is estimated at approximately 760 (Galletti Vernazzani et al. 2017). Chilean blue whales migrate seasonally between this foraging ground and the Eastern Tropical Pacific (Hucke-Gaete et al. 2018). In the Corcovado Gulf region of Northern Chilean Patagonia, wind interacts with freshwater input and tidal flux to generate regional productivity (Buchan et al. 2021), and blue whales feed on *Euphausia valentini* krill (Buchan and Quiñones 2016).

All three focal blue whale populations rely on productive coastal foraging grounds with different oceanographic characteristics, where they feed on different krill species. We hypothesize that the migratory life history of the ENP and Chilean populations may be facilitated, in part, by highly seasonal dynamics of the physical and biological oceanography that regulate prey availability on their foraging grounds. In contrast, the year-round residency of the New Zealand population may relate to the year-round availability of prey resources, though the scale of wind forcing and primary productivity is comparatively lower than the other two systems. Furthermore, we expect that the body shape of the migratory populations will be more streamlined to facilitate energetically efficient movement, for example, with narrower skulls and longer tails, compared to the resident New Zealand population. Morphological comparisons between blue whale populations in the context of oceanographic variability remain unexplored to date, presenting a key knowledge gap. Climate change is impacting marine species on a global scale (Poloczanska et al. 2013; Sydeman et al. 2015), while simultaneously driving locally extreme events such as marine heatwaves that are becoming more frequent and more intense with ocean warming (Oliver et al. 2018). Additionally, all three focal regions are subject to anthropogenic pressures such as shipping traffic (McKenna et al. 2015; Bedriñana-Romano et al. 2021), petroleum and mineral exploration (Torres 2013), or aquaculture infrastructure (Quiñones et al. 2019). Therefore, examining the interplay between the life history, morphology, and ecology of these blue whale populations and their environment is important for exploring the potential impacts of human activity and climate change.

In this study, we (1) compare regional oceanography between Monterey Bay, the STB, and the Corcovado Gulf by examining seasonality in physical forcing and biological productivity; and (2) compare the morphology of the ENP, New Zealand, and Chilean blue whale populations using aerial images collected using unoccupied aircraft systems (UAS). We hypothesize that variation in oceanography across ecosystems contributes to differences in morphology and migratory patterns between blue whale populations, and we draw on our findings in these focal study regions and the scientific literature to consider how variable environmental conditions relate to differences in migratory and feeding strategies among blue whale populations. Finally, we consider what these different life history strategies may imply for these populations in the face of climate change.

## Materials and methods

### Oceanography

We compared patterns of physical forcing that drive regional oceanography over a 10-year period using region-specific indices with documented importance to blue whales. In Monterey Bay, upwelling has been related to aggregation of forage species (Benoit-Bird et al. 2019) and blue whale feeding opportunities (Ryan et al. 2022). We downloaded the daily coastal upwelling transport index (CUTI), which estimates vertical flux off the United States West Coast (Jacox et al. 2018), for Monterey Bay (latitude 37º) between January 1, 2010 and December 31, 2019. In the STB, we used the upwelling index established by Chiswell et al. (2017), which is calculated as a difference in sea surface temperature (SST) between nearshore and offshore locations, and is a useful predictor of blue whale habitat (Barlow and Torres 2021). Daily SST was downloaded from the Multi-scale Ultra-high Resolution (MUR) satellite product to compute the upwelling index. In the Corcovado Gulf, zonal wind stress is related to zooplankton aggregation and blue whale foraging calls (Buchan et al. 2021). Daily zonal wind stress was downloaded from the Advanced Scatterometer (ASCAT METOP-A) satellite product. The zonal component describes the magnitude of east-west wind, which was averaged across the area spanning latitude −42º to −45º and longitude −73º to −75º. For upwelling index in the STB and wind stress in the Corcovado Gulf, annual patterns beginning 1 January would arbitrarily split the summer foraging season between calendar years. Therefore, the annual cycle was calculated beginning 1 July for Southern Hemisphere locations, so the 10-year period spanned July 1, 2009–June 30, 2019.

We generated a daily cumulative sum of the physical forcing metric for each year in each region. We then calculated a 10-year climatological mean for each cumulative physical measurement to represent the average seasonal cycle in physical forcing in each region. We characterized the degree of seasonality by computing 50 and 80% annual accumulation windows for each physical forcing metric. The 50% accumulation window represents the time between when the climatological mean reaches 25% and when it reaches 75% of the maximum annual accumulation value. Similarly, the 80% accumulation window represents the time between 10 and 90% of the maximum value. Therefore, in a system with relatively constant physical forcing throughout the year, we expect the climatological mean to increase linearly and the 50% accumulation window to span the middle portion of the year. However, in a highly seasonal system where most physical forcing occurs during a limited time of year, we expect the climatological mean to increase steeply within a narrow 50% accumulation window.

As a proxy for local biological productivity, we extracted monthly chlorophyll-*a* (Chl-*a*) concentration from the Visible Infrared Imaging Radiometer Suite (VIIRS) satellite product (Wang and Son 2016) over a 3º latitude x 3º longitude area in each region (Supplementary Fig. S1). VIIRS data are available beginning January 2012 and were downloaded through December 2019. We calculated a climatological mean of Chl-*a* for each region. Unlike the physical metrics, annual patterns in biological productivity can be directly compared between Monterey Bay, the STB, and the Corcovado Gulf. We therefore evaluated differences in total productivity and the degree of annual variation among the three regions.

Because freshwater input influences productivity in the Corcovado Gulf (Iriarte et al. 2017; Cuevas et al. 2019), we examined the relationship between sea surface salinity (SSS) and Chl-*a*. SSS data were obtained from the Aquarius satellite product, available 2011–2015. Since VIIRS data begin in 2012, this exploration was limited to 2012–2015. Daily SSS and Chl-*a* were averaged over the same spatial area (Supplementary Fig. S1) and compared visually and statistically using Pearson's correlation coefficient.

All satellite data were accessed via the ERDDAP server (https://coastwatch.pfeg.noaa.gov/erddap) using the R package “rerddapXtracto” (Mendelssohn 2019).

### Blue whale morphology

#### UAS data collection

Morphological measurements of blue whales were taken from aerial images collected using UAS. For the ENP population, flights over blue whales were conducted with a Mikrokopter LemHex-44 and FreeFly Alta 6 hexacopters between August 13, 2017 and August 16, 2017 and August 25, 2018 and September 5, 2018 in Monterey Bay (details in Bierlich, Hewitt et al. 2021). For the New Zealand population, UAS data were collected in the STB using DJI Phantom 3 Pro and DJI Phantom 4 Std quadcopters between February 2, 2016 and February 8, 2016 and February 8, 2017 and February 20, 2017 (details in Burnett et al. 2018; Torres et al. 2020). For the Chilean blue whale population, aerial images were collected using an APH-22 hexacopter in the Corcovado Gulf between February 22, 2015 and March 8, 2015 and February 23, 2017 and February 24, 2017 (details in Durban et al. 2016; Leslie et al. 2020). While each UAS contained an onboard barometer for recording altitude, beginning in 2017 each hexacopter was also fitted with a LightWare SF11/C laser altimeter that was co-located with the camera on a two-axis gimbal to ensure images and altitude were collected at nadir (Durban et al. 2015; Bierlich, Schick et al. 2021).

#### Photogrammetry and uncertainty quantification

**Fig. 2:**
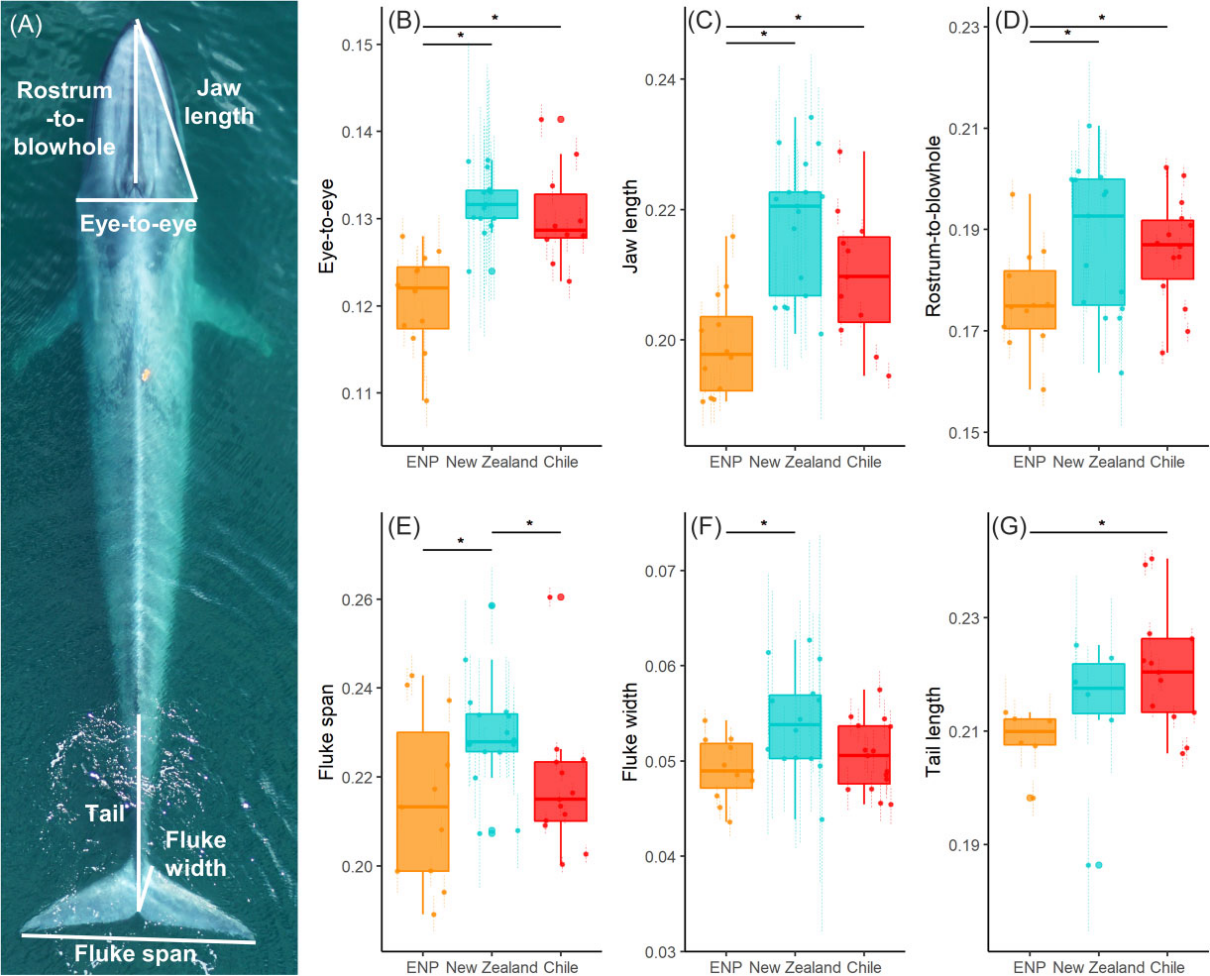
(A) Example UAS image of a blue whale, with morphological measurements depicted in white. (B–G) Each panel compares a specific morphological measurement between the ENP, New Zealand, and Chilean blue whale populations. Box plots show group median, interquartile range (IQR, box), maximum and minimum 1.5*IQR (vertical lines). Each point represents a measurement for an individual whale, with vertical lines showing the 95% HPDI. All measurements are standardized by total body length. Black horizontal bars with asterisks indicate statistically significant differences between groups based on Monte Carlo ANOVA results.

We used MorphoMetriX and CollatriX open source photogrammetry software (Bird and Bierlich 2020; Torres and Bierlich 2020) to measure the total length (TL, tip of rostrum to fluke notch), perpendicular widths in 5% increments of TL, rostrum-to-blowhole (RB), jaw length (JL), eye-to-eye (EE, also called bizygomatic width), tail length (Tail, posterior margin of dorsal fin to fluke notch), fluke span (Fs, a proxy for fluke size), and fluke width (Fw) (Fig. 2, Supplementary Fig. S2). We standardized morphological measurements (RB, JL, EE, Tail, and Fs) by TL for each whale.

We measured each whale's body condition using body area index (BAI), a scale-invariant, unitless metric with high precision and low uncertainty (Bierlich, Hewitt et al. 2021). BAI is calculated as the surface area across a range of the body length, standardized by TL (Burnett et al. 2018). Baleen whales deposit lipid stores primarily in the blubber (Brodie 1975), and BAI effectively captures changes in body condition over a foraging season in humpback (Bierlich et al. 2022) and gray (Soledade Lemos et al. 2020) whales, indicating increasing blubber stores from feeding. We calculated BAI using a head-tail range of 20–90% widths (Bierlich, Hewitt et al. 2021).

Each UAS image was ranked for measurability of TL and body condition following Christiansen et al. (2018). Additionally, images received a quality score of 1 (good), 2 (medium), or 3 (poor) for each morphological measurement (RB, JL, EE, Tail, Fs, and Fw). Images scored as 3 were removed from analysis. We also removed known calves (two in New Zealand and two in Chile) to avoid bias from smaller individuals. Up to five high-quality UAS images per whale were used for the ENP dataset, one image per whale for New Zealand (except two whales had two images), and one image per whale for the Chilean dataset. Based on individual photo-identification conducted concurrently during fieldwork, we determined that no individual blue whales were measured in multiple years in any of the study regions. In total, the final dataset contained 31 ENP, 17 New Zealand, and 15 Chilean blue whales.

To account for photogrammetric uncertainty associated with each UAS, we applied a Bayesian statistical model for observation errors. Rather than a single point estimate, the Bayesian statistical model uses measurements of known-sized objects in UAS images as training data to generate a posterior predictive distribution for each morphological measurement (Bierlich, Schick et al. 2021). For the APH 22, we used measurements of the research vessel as training data (18.62 m, Durban et al. 2016; Leslie et al. 2020). For the Alta and LemHex, we used known sized objects floating at the surface (1.48 m, 1.27 m, Bierlich, Schick et al. 2021). For the P4S we used measurements of known-sized objects on the research vessel and floating at the surface (4.41 m, 2.00 m, Burnett et al. 2018).

#### Statistical analysis

We used Analysis of Variance (ANOVA) to compare each morphological measurement (TL, BAI, RB, EE, JL, Tail, Fs, and Fw) between populations, where population is the predictor variable, and the morphological measurement is the response variable. We used Monte Carlo methods to propagate photogrammetric uncertainty associated with each UAS by averaging the results of 1,000,000 replications of the ANOVA for each morphological measurement (Torres et al. 2022). For each replicate, we sampled each whale's morphological measurement (e.g., RB) from a normal distribution parameterized with the posterior mean and variance from that whale's posterior distribution for that morphological measurement. We also calculated the difference between the coefficients for each morphological measurement of each population. The Monte Carlo average coefficients, difference between population coefficients, and Highest Posterior Density Intervals (HPDI) were calculated for each ANOVA.

To examine the scaling of different morphological measurements with TL, we fitted a linear regression between the point estimates for each morphological measurement and TL on a log-log scale. A slope of 1 indicates isometry (i.e., proportional scaling with body length), whereas a slope above or below 1 indicates either positive or negative allometry. Prior to fitting each linear regression, we tested for the presence of outliers using a Dixon's Q test, which is well suited for small sample sizes (Dixon 1953), so that any potential bias or skew could be removed *a priori*.

All analyses were conducted in R (R Core Team 2019).

## Results

### Oceanography

Both the STB and Corcovado Gulf exhibited relatively little seasonality of physical forcing, based on the 10-year climatological means (Fig. 1). Cumulative upwelling index for the STB increased steadily throughout the year, with the 50% accumulation spanning 191 days (September 23–April 1). Cumulative zonal wind stress in the Corcovado Gulf increased steadily, and the 50% accumulation window was similar in length to the STB but slightly earlier, spanning 190 days (September 13–March 23). In contrast, the seasonality in physical forcing was more pronounced in Monterey Bay, where the 50% accumulation window for CUTI spanned 120 days (April 29–August 27).

The Corcovado Gulf had the highest primary productivity of the three regions, with moderate productivity in Monterey Bay, and the lowest productivity in the STB (Fig. 1). The Corcovado Gulf also exhibited the strongest seasonality in productivity, illustrated by a clear annual cycle in Chl-*a* peaking in January. Additionally, a significant negative correlation between SSS and Chl-*a* across the Corcovado Gulf indicated that lower salinities correspond with higher productivity (Pearson's correlation coefficient = −0.42, *P* < 0.001; Supplementary Fig. S3). While seasonality in productivity was weaker for Monterey Bay and the STB than the Corcovado Gulf, mean annual productivity had a slightly bimodal shape for both, with peaks in spring and fall (Fig. 1).

In summary, oceanographic analyses revealed that Monterey Bay exhibited the highest physical seasonality, moderate biological seasonality, and moderate biological productivity. The Corcovado Gulf displayed low physical seasonality but high biological seasonality, and the highest biological productivity. The STB had both comparatively low physical and biological seasonality, and the lowest biological productivity among the regions (Fig. 1).

### Blue whale morphology

UAS flights were conducted in late summer-early fall in each region, with slightly earlier sampling in the STB than Monterey Bay or the Corcovado Gulf (Fig. 1). Mean TL was 22.36 m (95% HPDI = 21.79, 22.96 m) for the ENP population, 21.87 m (20.87, 22.88 m) for the New Zealand population, and 21.93 m (21.08, 22.77 m) for the Chilean population. There was no significant difference in TL between populations (Fig. 1, Table 1, Supplementary Table S1, Supplementary Fig. S4). Despite indistinguishable body lengths between populations and similar timing of UAS sampling between regions, body condition differed significantly. Specifically, the New Zealand population had significantly higher BAI than either the ENP or Chilean population (Fig. 1, Table 1, Supplementary Table S1, Supplementary Fig. S4).

**Table 1:** Results from Monte Carlo ANOVA analysis (1,000,000 replications), showing average difference between morphological measurements of each population. The lower and upper 95% HPDI are shown in parentheses. Comparisons where HPDI do not overlap 0 are considered statistically significant, denoted by asterisks.

Measurement	New Zealand-ENP	New Zealand-Chile	ENP-Chile
Total length	−0.49 (−1.654, 0.668)	−0.054 (−1.371, 1.256)	0.436 (−0.593, 1.466)
Body area index	1.34 (0.883, 1.843)*	1.684 (0.982, 2.365)*	0.345 (−0.223, 0.949)
Eye-to-eye	0.011 (0.006, 0.016)*	0.002 (−0.004, 0.007)	−0.01 (−0.015, −0.005)*
Jaw length	0.018 (0.01, 0.027)*	0.008 (−0.001, 0.016)	−0.011 (−0.019, −0.002)*
Rostrum-to-blowhole	0.012 (0.002, 0.022)*	0.002 (−0.008, 0.012)	−0.01 (−0.02, 0)*
Fluke span	0.015 (0.002, 0.027)*	0.011 (0, 0.023)*	−0.003 (−0.016, 0.01)
Fluke width	0.005 (0, 0.01)*	0.004 (−0.001, 0.008)	−0.001 (−0.006, 0.003)
Tail length	0.005 (−0.008, 0.019)	−0.007 (−0.019, 0.004)	−0.001 (−0.023, −0.001)*

**Fig. 3:**
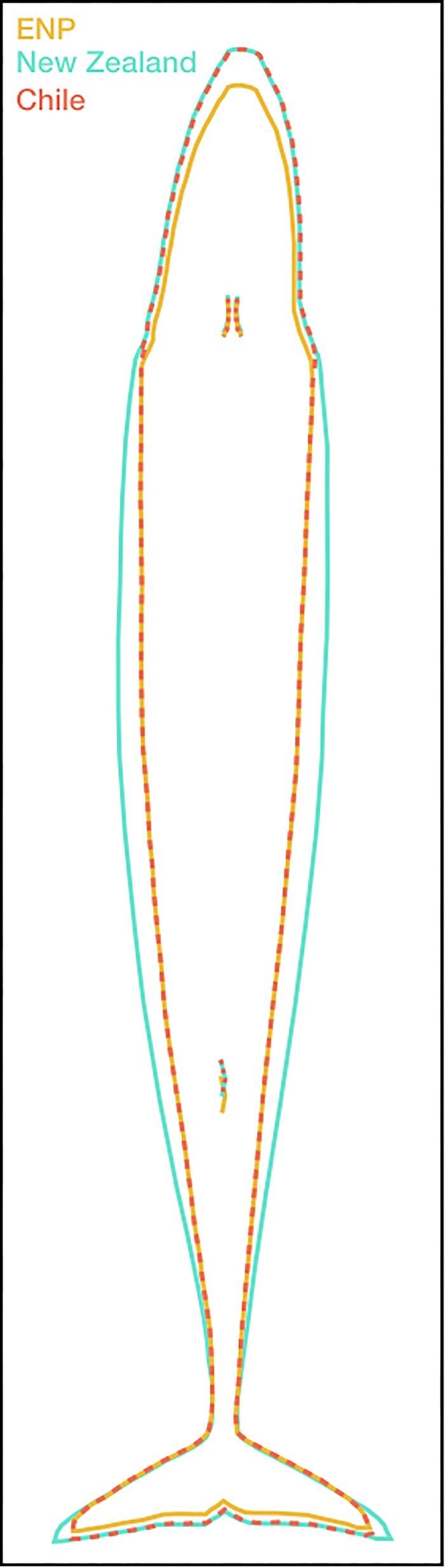
Scaled schematic comparing morphology between the ENP, New Zealand, and Chilean blue whale populations. Separated lines indicate statistically significant differences; overlapping dashed lines indicate no significant differences.

**Fig. 4:**
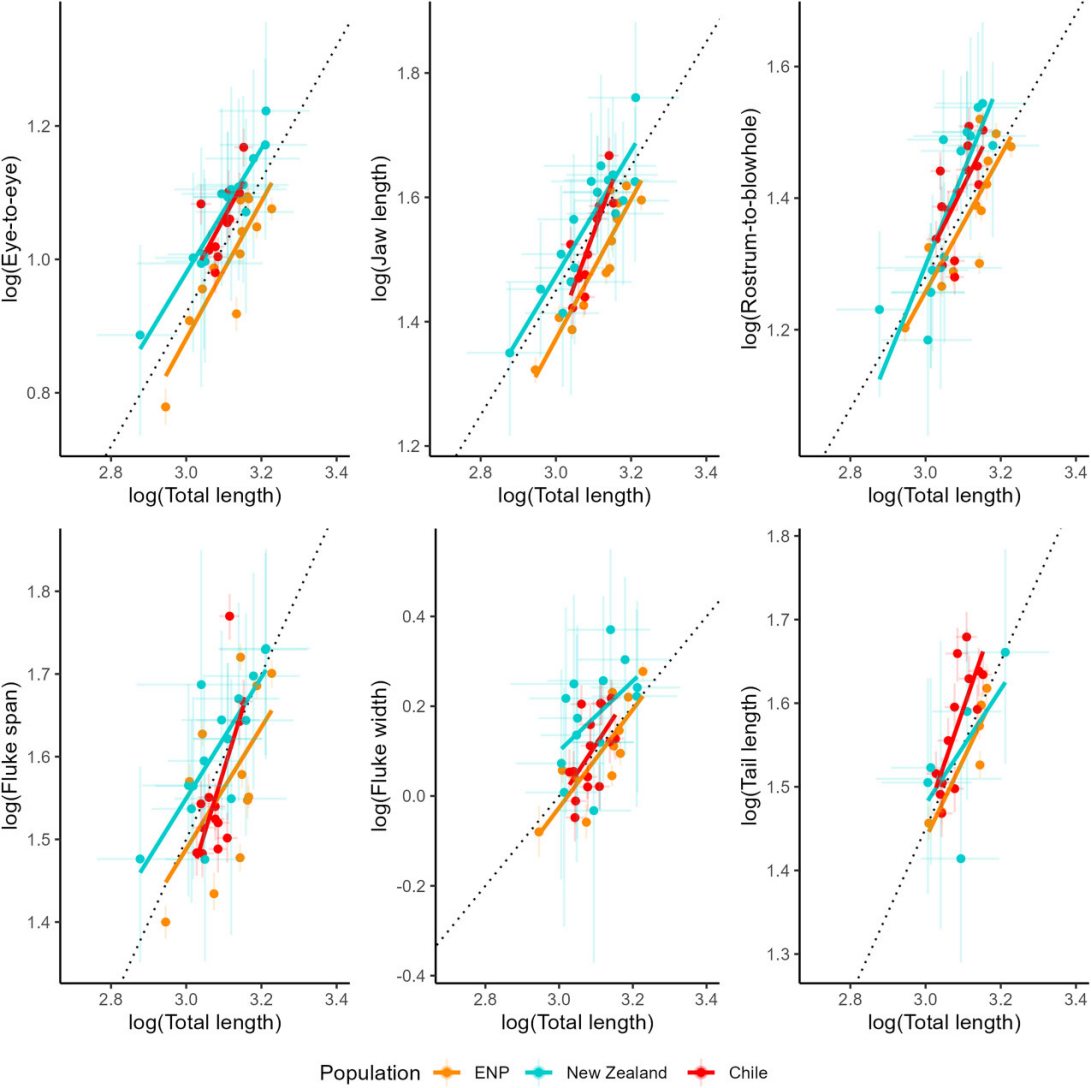
Scaling of each morphometric measurement compared to body length, on a log-log scale. Dotted black lines represent a theoretical isometric relationship, where slope = 1. Each point represents an individual blue whale. Light bars around each point represent the 95% HPDI associated with each measurement. Solid, colored lines show fitted linear relationships by population (resulting coefficients in Supplementary Table S2).

ENP blue whales had smaller skulls than either the New Zealand or Chilean populations, with significantly smaller EE, JL, and RB measurements. The New Zealand population had the largest flukes, with significantly greater Fs than either the ENP or Chilean population and significantly wider Fw than the ENP population. The Chilean population had the longest Tail measurement, significantly greater than the ENP population (Fig. 2, Fig. 3, Table 1, Supplementary Table S1, Supplementary Fig. S5). The Dixon's test did not identify any outliers in any of the morphometric measurements for any of the three populations; therefore, all measurements were retained for further analysis. We generally observed slight positive allometry between TL and skull morphology (EE, JL, and RB) in all three populations. Fluke morphology (Fw, Fs, and Tail) showed strong positive allometry for the Chilean population, but strong negative allometry for the New Zealand population; ENP fluke morphology showed negative allometry in Fs only (Fig. 4, Supplementary Table S2).

Overall, morphological comparisons showed that while all three populations are comparable in TL, the New Zealand population had the largest skulls and flukes, and the highest body condition. The ENP population had smaller skulls and flukes than the other two populations, and low body condition compared to New Zealand. The Chilean population had large skulls, the longest tails, and low body condition compared to New Zealand.

## Discussion

We document how three blue whale populations, representing three subspecies, differ morphologically despite being indistinguishable in length. Our analyses of physical and biological oceanography of their foraging grounds illustrate variable patterns of seasonality and productivity that regulate blue whale foraging opportunities. We show that in the more seasonal and productive Monterey Bay and Corcovado Gulf foraging grounds, blue whales were in lower body condition than in the less productive and less seasonal STB. Similar to previous work relating animal morphology to ecology and life history patterns at the species level (Webb 1984; Aerts et al. 2000; Woodward et al. 2006; Pigot et al. 2020), we integrate these results below to describe how, and hypothesize why, the form of each blue whale population may be “shaped by their environment.” Furthermore, the contrasting migratory phenology, morphology, and feeding behaviors of these three blue whale populations may have implications for their relative vulnerability to changing ocean conditions.

### Feeding ground seasonality, blue whale phenology, and body condition

Seasonality of foraging grounds may be an important factor driving differences in blue whale life history and migratory phenology. In Monterey Bay, a defined upwelling season (Fig. 1) leads to an annual spring bloom in productivity and prey for blue whales (Croll et al. 2005). Furthermore, within-season physical forcing aggregates krill on shorter timescales (Benoit-Bird et al. 2019), enabling blue whales to capitalize on this ephemeral prey resource (Cade et al. 2021; Ryan et al. 2022). In the Corcovado Gulf, seasonality is less defined by wind, but the influence of freshwater yields a strong annual cycle in primary productivity (Fig. 1, Supplementary Fig. S3) (Cuevas et al. 2019). Areas of high Chl-*a* drive blue whale habitat use in the Corcovado Gulf (Bedriñana-Romano et al. 2018), and krill are twice as abundant in spring-summer compared to winter (Caruso et al. 2021). In contrast, the STB region exhibits comparatively low seasonality in both physical forcing and surface Chl-*a* (Fig. 1), and year-round pulsed upwelling events (Chiswell et al. 2017) and blue whale occurrence patterns (Barlow et al. 2023) indicate that blue whale foraging opportunities may exist throughout the year, though at a lower magnitude than the other regions. Given that blue whales are highly mobile and are known to track resource availability throughout their foraging range (Abrahms et al. 2019), we acknowledge that the regions for which we examine oceanographic seasonality represent a portion of the broader foraging range accessible to each population, particularly the migratory ENP (Calambokidis et al. 2009; Bailey et al. 2010) and Chilean (Torres-Florez et al. 2014; Bedriñana-Romano et al. 2018) populations. However, Monterey Bay (Calambokidis et al. 2015), the Corcovado Gulf (Galletti Vernazzani et al. 2017), and the STB (Barlow et al. 2018) are consistently important foraging grounds for each population within their range and represent the feeding locations where morphometric data were collected. We acknowledge the broader geographic context of foraging for each population, yet we assume these three focal study areas are representative of oceanographic and prey conditions affecting the measured whales, and therefore relevant for the comparisons made in this study.

The ENP and Chilean blue whale populations that migrate away from their highly seasonal foraging grounds must synchronize their movements with ecosystem phenology (Szesciorka et al. 2020; Oestreich et al. 2022), and rely on memory from previous years to exploit seasonally abundant prey (Abrahms et al. 2019). While ENP and Chilean blue whales feed throughout their range to some extent (Hucke-Gaete et al. 2018; Busquets-Vass et al. 2021), timing their presence on the foraging grounds is critical for acquiring enough energy to support their migratory life history strategy. Although these highly seasonal, highly productive foraging grounds present an advantage in abundant prey years, long-distance migration to these areas also presents a risk if the timing is not aligned, or if productivity is low. The comparatively lower body condition of ENP and Chilean blue whales implies that migratory populations may be more vulnerable to unpredictable environmental conditions, which are anticipated under climate change (Frölicher et al. 2018). However, some projections for upwelling systems under climate change include longer and more intense upwelling seasons (Sydeman et al. 2014; Wang et al. 2015), which could create favorable foraging conditions for migratory blue whales in eastern boundary current upwelling regions such as Monterey Bay. Furthermore, migratory blue whales are known to have the behavioral flexibility to capitalize on years with longer, more intense upwelling (Oestreich et al. 2022). In contrast, the strategy of remaining in a lower-productivity foraging area year-round, as employed by the New Zealand population, may enable exploitation of relatively lower amounts of prey, but with more consistent energetic benefits over a longer annual period. In years with morphological data, the significantly higher body condition of the New Zealand population (Fig. 1, Table 1, Supplementary Fig. S4) indicates the advantage of their strategy. Body condition of baleen whales is known to vary considerably within and between years (Soledade Lemos et al. 2020; Bierlich et al. 2022), and we acknowledge that the limited number of years and relatively small sample sizes contained in this study likely represent only a snapshot of broader-scale and longer-term patterns. These hypotheses could be further tested by comparing body condition of migratory (ENP, Chilean) and non-migratory (New Zealand) populations across longer time periods.

### Morphology and foraging behavior

Morphological differences between blue whale populations may relate to different feeding behaviors, driven by differences in prey characteristics between foraging grounds. In Monterey Bay, blue whales feed predominantly on dense krill aggregations (*T. spinifera* and *E. pacifica*) at depths of 100–300 m (Hazen et al. 2015), and their dives track the diel vertical migration of their prey (Croll et al. 2001). In contrast, the *N. australis* krill found in New Zealand often swarm at the surface, and generally lack diel or depth patterns relative to biomass (O'Brien 1988; Young et al. 1993). As a result, it is likely energetically favorable for New Zealand blue whales to feed on krill aggregations at the surface (Torres et al. 2020). The Corcovado Gulf seems to be an intermediate scenario between the deep prey in Monterey Bay and shallow prey in New Zealand, whereby *E. valentini* have diel vertical migration and blue whales dive to intermediate depths (average 30 m; highly variable) for foraging (Caruso et al. 2021).

In terms of skull and fluke morphology, the New Zealand and Chilean populations are most similar, while the ENP and New Zealand populations have the strongest differences (Fig. 2, Fig. 3, Table 1, Supplementary Fig. S5). The smaller skulls of ENP blue whales may be better-suited for deep foraging and long-distance movement by reducing drag forces (Gough et al. 2022). The robust morphology of the non-migratory New Zealand blue whales may be advantageous for overcoming decreased swimming efficiency during surface-feeding due to increased buoyancy from higher blubber reserves (Aoki et al. 2021); larger flukes could increase propulsion and larger skulls may facilitate greater engulfment capacity (Kahane-Rapport and Goldbogen 2018), ultimately improving foraging efficiency at the surface. Chilean blue whales fall between the two, with long tails and large skulls. Perhaps this morphology of Chilean blue whales reflects their long-range migration, and supports surface feeding while in the Corcovado Gulf (Buchan and Quiñones 2016; Caruso et al. 2021).

The scaling of morphological features with body length revealed isometry or slight positive allometry in skull dimensions for all three populations. This pattern supports previously described measurements of blue whales from whaling records, and aligns with similar allometric scaling of skull dimensions in other large rorqual whale species (Kahane-Rapport and Goldbogen 2018). Fluke morphology showed contrasting patterns between populations; notably positive allometry in tail length for the Chilean population but negative allometry for the New Zealand population. The subspecies name “*brevicauda*” for pygmy blue whales, including the New Zealand population, is descriptive of their shorter tail compared to Antarctic blue whales (Ichihara 1966). The longer tail of the Chilean population compared to pygmy blue whales has provided evidence for their classification as a distinct subspecies (Leslie et al. 2020). Despite tail length being used as a defining morphological feature in subspecies delineation (Ichihara 1966; Leslie et al. 2020; Pastene et al. 2020), the exact locomotory trade-offs of differences in tail lengths are unclear; however, a longer tail could enable higher-amplitude heave in the tailbeat cycle (Gough et al. 2021; Gough et al. 2022). While we did not necessarily find clear differences in tail morphology by comparing population level means, differences may be more apparent in the population-specific allometric scaling (Fig. 4, Supplementary Table S2).

We note that the morphological differences we describe are subtle compared to differences between baleen whale species (Woodward et al. 2006; Gough et al. 2019); nevertheless, our findings illustrate a morphological spectrum across populations within a single species. While we present hypotheses regarding functional drivers of morphology based on foraging differences, these remain to be tested empirically in future research. Furthermore, we acknowledge that our comparisons do not consider the genetic relatedness of the three subspecies, which may yield further insights. Undoubtedly, there are additional factors at play influencing blue whale morphology that interact with the seasonal oceanography and prey availability on the different feeding grounds, and thus behavioral differences between regions as described in this study.

### Blue whales in a changing ocean

In all three study regions, blue whales contend with anthropogenic pressures on their foraging grounds. In California, vessel traffic generates collision risk and noise (McKenna et al. 2015; Redfern et al. 2017). The Corcovado Gulf foraging grounds likewise overlap with a major vessel traffic corridor (Bedriñana-Romano et al. 2021), and are the site of a growing aquaculture industry that further increases vessels, influences nutrient cycling, and creates entanglement risk (Quiñones et al. 2019). In the STB, petroleum exploration and extraction represents the predominant human activity (Torres 2013; Barlow & Torres 2021); however, the STB is likely less impacted by human pressures compared to the other regions (i.e., less shipping traffic density). Body condition in baleen whales is linked to stress physiology (Lemos et al. 2021; Pirotta et al. 2023), and the lower body condition of the ENP and Chilean populations may relate to elevated stressors (environmental or anthropogenic) in those regions compared to New Zealand. Our findings are based on limited sample sizes and years, and thus represent a subset of the blue whales and the environmental variability in each region. The ENP and Chilean populations may display higher body condition following periods of stronger physical forcing and higher productivity. Future data collection enabling more detailed analyses of inter- and intra-seasonal variation in body condition is therefore required to elucidate how blue whales gain and allocate energy under variable environmental conditions. As ocean temperatures rise due to climate change and human exploitation of ocean ecosystems increases, more research is needed on the resilience or vulnerability of these populations.

## Supplementary Material

obad039_Supplemental_File

## Data Availability

The processed datasets generated for this study and relevant analysis code can be accessed via the following Figshare Digital Repository: https://doi.org/10.6084/m9.figshare.24282724.v2
