# Supplementary material for: Shaped by Their Environment: Variation in Blue Whale Morphology across Three Productive Coastal Ecosystems
Source: Integr Org Biol. 2023 Nov 20;5(1):obad039. doi: 10.1093/iob/obad039 (PMC10701340; doi:10.1093/iob/obad039)
Supplement: obad039_Supplemental_File [file obad039_supplemental_file.pdf]

Shaped by their environment: variation in blue whale morphology across three productive coastal ecosystems

Dawn R. Barlow[1]*, K.C. Bierlich[1], William K. Oestreich[2], Gustavo Chiang[3], John W. Durban[4], Jeremy A. Goldbogen[5], David W. Johnston[6], Matthew S. Leslie[7], Michael Moore[8], John P. Ryan[2], Leigh G. Torres[1]

[1]Geospatial Ecology of Marine Megafauna Lab, Marine Mammal Institute, Department of Fisheries, Wildlife, and Conservation Sciences, Oregon State University, Newport, Oregon USA

[2]Monterey Bay Aquarium Research Institute, Moss Landing, California, USA

[3]Centro de Investigación para la Sustentabilidad (CIS) & Departamento de Ecología y Biodiversidad, Universidad Andrés Bello, Santiago, Chile

[4]Marine Mammal Institute, Oregon State University, Newport, Oregon, USA

[5]Hopkins Marine Station, Department of Biology, Stanford University, Pacific Grove, California, USA

[6]Division of Marine Science and Conservation, Nicholas School of the Environment, Duke University Marine Laboratory, Beaufort, North Carolina, USA

[7] National Climate Adaptation Science Center, United States Geological Survey, Reston, Virginia, USA

[8]Biology Department, Woods Hole Oceanographic Institution, Woods Hole, Massachusetts, USA

*dawn.barlow@oregonstate.edu

SUPPLEMENTARY MATERIALS

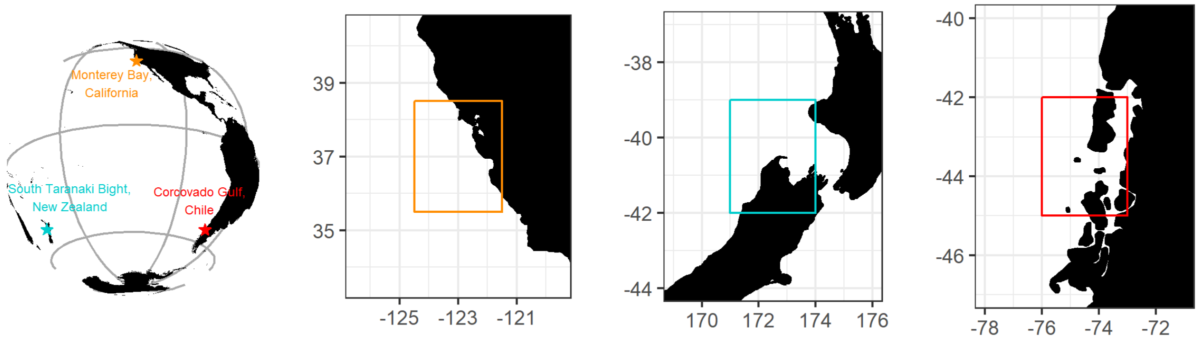

Figure S1. Areas over which monthly mean VIIRS Chl-*a* satellite data were extracted in each of the three regions examined.

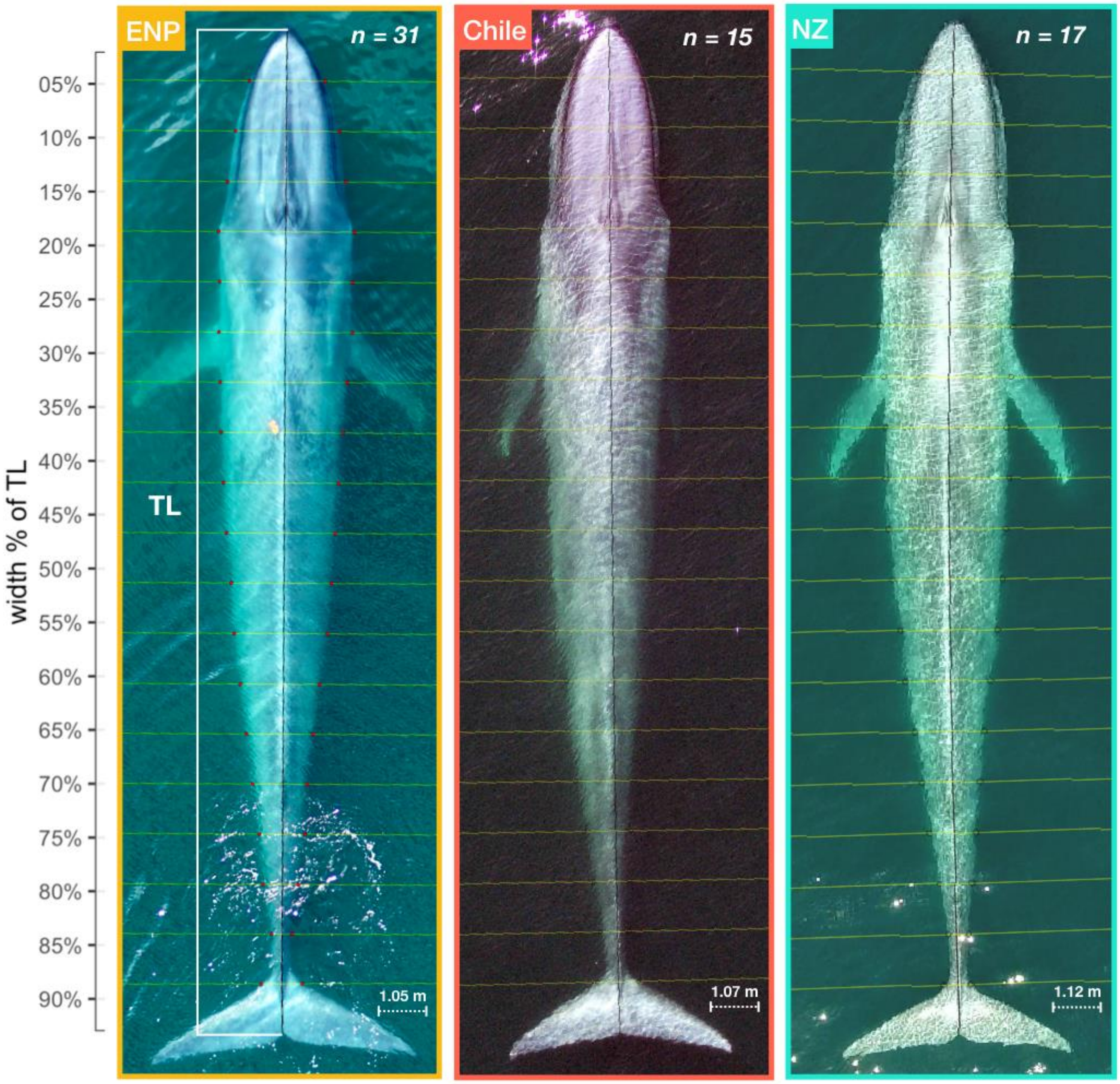

Figure S2. Example of total length (TL) and width measurement outputs from MorphoMetriX software of Eastern North Pacific (ENP), Chile, and New Zealand (NZ) blue whales, with sample size for each population (*n*). A scalebar is included on each image for comparison. Total length measurements for the whales in the selected images are: ENP = 20.97 m (20.71, 21.21), Chile = 21.33 m (20.73, 21.89), and NZ = 22.43 m (29.45, 24.59).

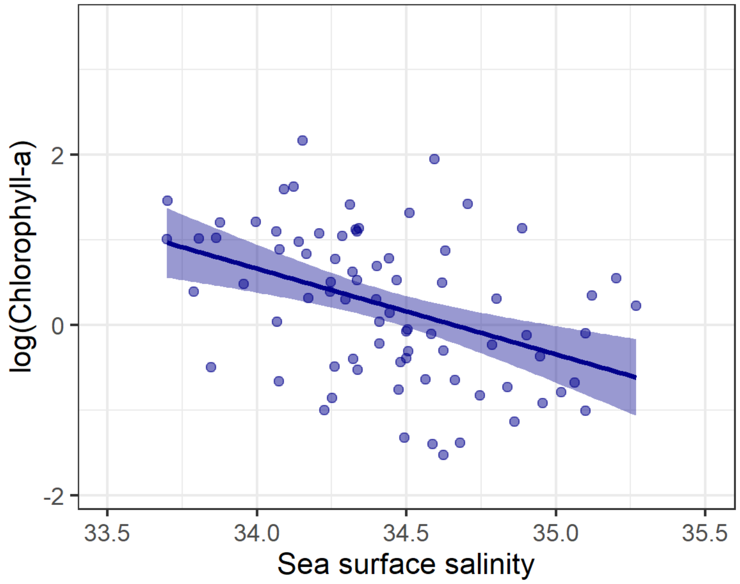

Figure S3. Correlation between sea surface salinity and chlorophyll-a concentration in the Corcovado Gulf between 2012-2015. Chlorophyll-a concentration was obtained from the VIIRS satellite product and salinity was obtained from the Aquarius satellite product, and both were averaged over the study area at a daily scale where measurements were available (Fig. S1).

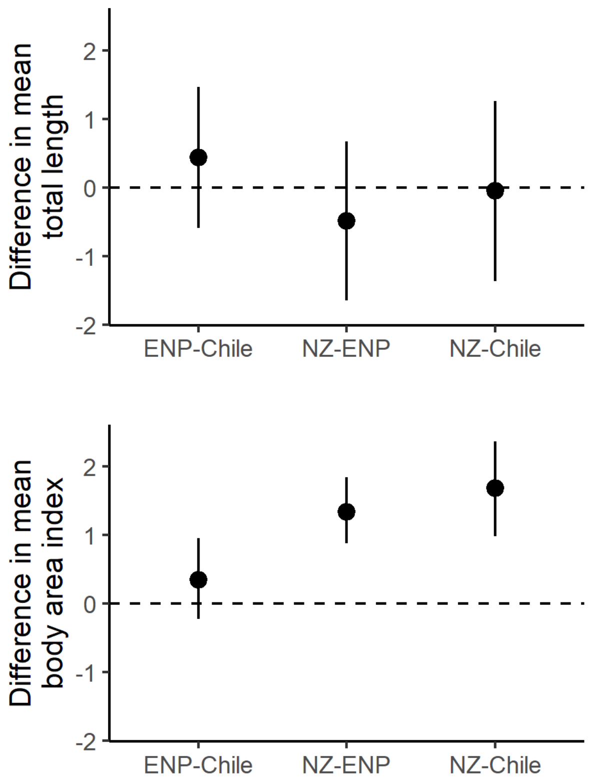

Figure S4. Results of the Monte Carlo ANOVAs (1,000,000 replications) comparing differences in mean total length and body area index between blue whale populations, Eastern North Pacific (ENP), New Zealand (NZ), and Chile. Each point represents the difference in means, and vertical lines represent 95% HPD intervals. Comparisons where the HPD interval lines cross the horizontal dashed line at zero indicate that the difference is not statistically significant.

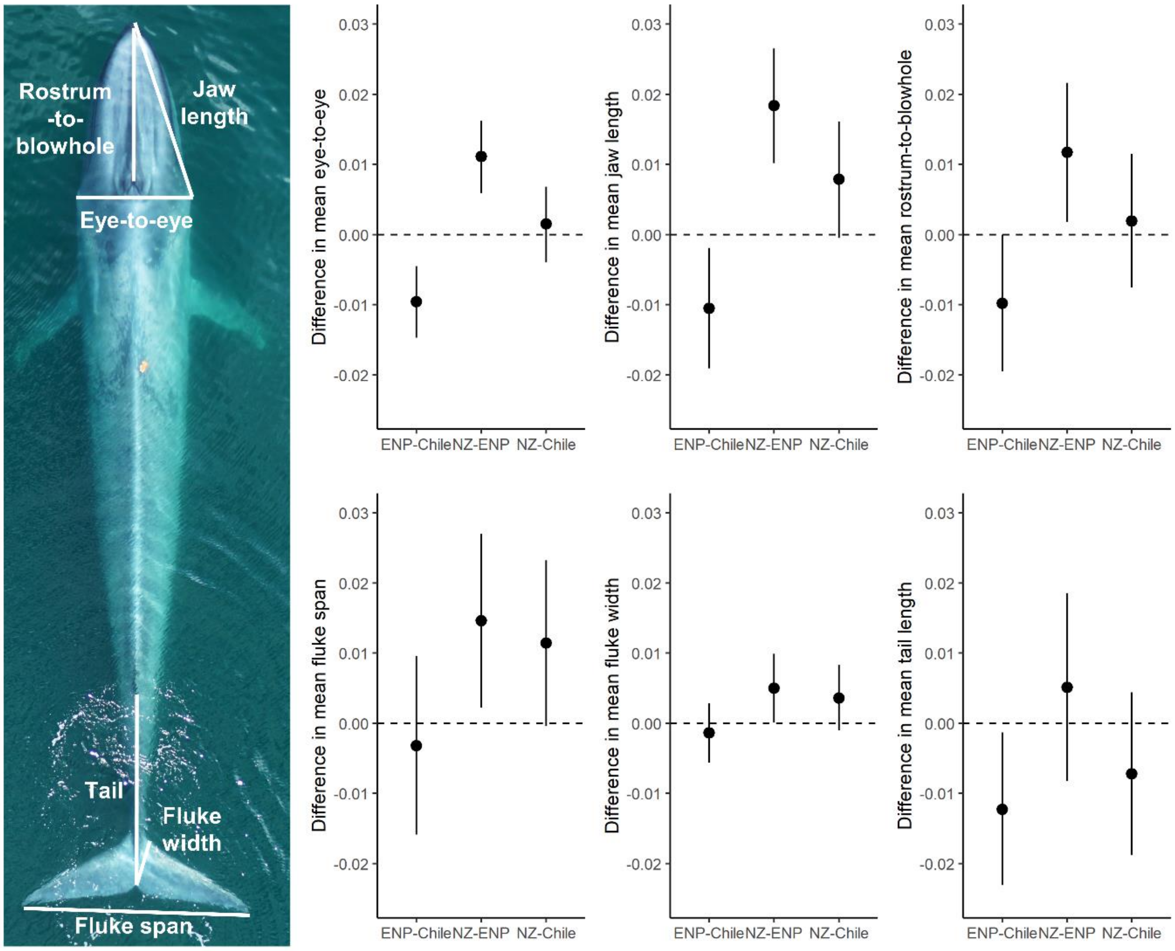

Figure S5. Results of the Monte Carlo ANOVAs (1,000,000 replications) comparing differences in morphological measurements between blue whale populations, Eastern North Pacific (ENP), New Zealand (NZ), and Chile. Each point represents the difference in means, and vertical lines represent the 95% highest posterior density (HPD) intervals. Comparisons where the HPD interval lines cross the horizontal dashed line at zero indicate that the difference is not statistically significant.

Table S1. The average coefficients for each morphological measurement of each population from Monte Carlo ANOVA results (1,000,000 replications). The lower and upper 95% highest posterior density (HPD) intervals are shown in parentheses. All measurements are standardized by total length (TL).

Measurement	New Zealand	Chile	Eastern North Pacific
Total length (m)	21.88 (20.88, 22.88)	21.93 (21.09, 22.78)	22.37 (21.79, 22.96)
Body area index	14.36 (13.84, 14.77)	12.67 (12.21, 13.23)	13.02 (12.74, 13.33)
Eye-to-eye	0.13 (0.13, 0.14)	0.13 (0.13, 0.13)	0.12 (0.12, 0.12)
Jaw length	0.22 (0.21, 0.22)	0.21 (0.2, 0.22)	0.2 (0.19, 0.21)
Rostrum-to-blowhole	0.19 (0.18, 0.19)	0.19 (0.18, 0.19)	0.18 (0.17, 0.18)
Fluke span	0.23 (0.22, 0.24)	0.22 (0.21, 0.23)	0.21 (0.21, 0.22)
Fluke width	0.05 (0.05, 0.06)	0.05 (0.05, 0.05)	0.05 (0.05, 0.05)
Tail length	0.21 (0.2, 0.22)	0.22 (0.21, 0.23)	0.21 (0.2, 0.22)

Table S2. Scaling relationships between total body length and each of the morphological measurements examined. The slope, intercept, and r^2 values are the result of a linear model fitted for each scaling relationship, examined separately for the Eastern North Pacific (ENP), New Zealand, and Chilean blue whale populations. Relationships exhibiting positive allometry (slope > 1) are shown in blue, relationships exhibiting negative allometry (slope < 1) are shown in the red, and relationships that are generally isometric (slope ≈ 1) are shown in black. Sample sizes indicate the number of unique individual whales captured via UAS for each measurement.

	Measurement	Population	Slope	Intercept	r^2	Sample size
**Skull morphology**	Eye-to-eye	ENP	1.027	-2.200	0.738	12
		New Zealand	0.931	-1.814	0.903	15
		Chile	1.034	-2.144	0.353	10
	Jaw length	ENP	1.122	-1.994	0.834	12
		New Zealand	1.007	-1.548	0.767	17
		Chile	1.647	-3.564	0.642	11
	Rostrum-to-blowhole	ENP	1.035	-1.850	0.662	12
		New Zealand	1.413	-2.943	0.670	15
		Chile	1.152	-2.155	0.352	14
**Fluke morphology**	Fluke width	ENP	1.092	-3.302	0.621	11
		New Zealand	0.778	-2.235	0.175	14
		Chile	1.250	-3.760	0.275	15
	Fluke span	ENP	0.737	-0.724	0.276	11
		New Zealand	0.732	-0.648	0.606	16
		Chile	1.484	-3.018	0.416	13
	Tail length	ENP	0.942	-1.388	0.800	6
		New Zealand	0.687	-0.583	0.254	6
		Chile	1.305	-2.455	0.551	13
